# Persistent metabolic remodeling after dietary salt reduction reveals sex differences in the adaptation to chronic high-salt intake

**DOI:** 10.1186/s13293-026-01017-1

**Published:** 2026-11-12

**Authors:** Sevilay Şahoğlu Göktaş, Peter Spégel, Martin Magnusson, Anja Meissner, João M. N. Duarte

**Affiliations:** 1 Wallenberg Center for Molecular Medicine, Lund University, Lund, Sweden; 2 Department of Experimental Medical Science (EMV), Faculty of Medicine, Lund University, Sölvegatan 19, BMC C11, Lund, 221 84 Sweden; 3 Department of Chemistry, Centre for Analysis and Synthesis, Lund University, Lund, 223 62 Sweden; 4 Division of Physiology & Vascular Biology, Institute of Theoretical Medicine, Faculty of Medicine, University of Augsburg, Augsburg, Germany; 5 Department of Clinical Sciences, Lund University, Malmö, Sweden; 6 Department of Cardiology, Skåne University Hospital, Malmö, Sweden; 7 Hypertension in Africa Research Team (HART), North-West University, Potchefstroom, South Africa

**Keywords:** Hypertension, Cardiac remodeling, Metabolism, Dietary salt

## Abstract

**Background:**

Excessive salt intake is a risk factor for cardiovascular disease, yet its systemic metabolic consequences and its relationship to differential cardiovascular adaptations in males and females remain poorly understood. Here, we performed plasma metabolite profiling in male and female mice subjected to long-term high-salt diet (HSD) exposure during aging.

**Methods:**

Mice were fed HSD (8.6% NaCl) or low-salt normal diet (ND; 0.6% NaCl) from 9 to 23 months of age, with a subset of mice undergoing diet reversal to ND after 7 months of HSD. Plasma metabolites were analyzed using gas chromatography/mass spectrometry and related to previously reported measurements of arterial blood pressure and cardiac function.

**Results:**

Female mice developed salt-sensitive hypertension driven by vascular dysfunction, whereas male mice exhibited cardiac and vascular remodeling independent of blood pressure. Among 48 measured metabolites, HSD induced substantial alterations in levels of glucose, glycerol, glycerol phosphates, and fatty acids. Males showed a near-complete normalization of metabolite profiles following 7 months of salt reduction, whereas females retained a metabolomic signature different from ND despite normalization of blood pressure. In particular, metabolites related to energy metabolism, lipid handling, and osmotic balance were influenced by both diet and sex, with linoleate showing a significant sex-diet interaction. Correlation analyses demonstrated associations between metabolite profiles and cardiovascular parameters with different patterns in males and females.

**Conclusion:**

Our findings demonstrate that chronic high-salt consumption induces persistent plasma metabolite alterations displaying different patterns in male and female mice that are not fully reversed by a salt-lowering dietary intervention. The dissociation between physiological recovery and metabolic normalization, particularly in females, suggests that metabolic alterations may represent an additional layer of cardiovascular risk beyond blood pressure control in mice.

**Supplementary Information:**

The online version contains supplementary material available at https://doi.org/10.1186/s13293-026-01017-1.

## Abstract (plain language summary)

Eating too much salt is known to increase the risk of heart and blood vessel diseases, but its long-term effects on metabolism and whether these responses to high salt intake differ between males and females are less well understood. In this study, we examined a panel of blood metabolites in male and female mice after long-term consumption of a high-salt diet. We also investigated whether these metabolic changes could be reversed by returning the animals to a normal-salt diet. We found that males and females responded differently to prolonged salt intake. Although reducing dietary salt improved blood pressure in females and heart function in males, the underlying metabolic changes did not recover equally. Male mice showed an almost complete return to a normal metabolic profile, whereas females retained some of the metabolic alterations caused by the high-salt diet, even after blood pressure had returned to normal. These findings suggest that lowering dietary salt may not fully reverse the biological effects of long-term salt exposure, particularly in females. They also highlight that blood pressure alone may not reflect the full impact of excess salt on health. Understanding these sex differences may help improve strategies to prevent and treat cardiovascular disease through more personalized dietary and therapeutic approaches.

**Supplementary Information:**

The online version contains supplementary material available at https://doi.org/10.1186/s13293-026-01017-1.

## Study highlights

Chronic high-salt intake induces persistent remodeling of the circulating metabolome during aging with different patterns observed in males and females.

Dietary salt reduction restores the plasma metabolome more completely in males than in females, despite recovery of blood pressure in females.

Lipid metabolism is a major target of long-term salt exposure, with fatty acids emerging as key contributors to sex differences in metabolic adaptation.

Metabolic recovery can remain incomplete despite physiological improvement, suggesting that circulating metabolites provide additional information beyond conventional cardiovascular measurements.

**Supplementary Information:**

The online version contains supplementary material available at https://doi.org/10.1186/s13293-026-01017-1.

## Introduction

Excessive dietary salt intake is a major contributor to cardiovascular disease and is strongly associated with hypertension and end-organ damage. Considerable evidence links excess dietary salt intake to the development of hypertension, left ventricular hypertrophy, as well as to an increased risk of stroke and coronary heart disease [1, 2]. Pre-clinical and clinical data support that even in the absence of blood pressure increase, excess dietary salt can adversely affect target organs, including the blood vessels, heart, kidneys, and brain [3].

Amongst patients with essential hypertension, dietary salt consumption is a causative factor in 50–80% of cases [4]. The prevailing clinical notion is that a subset of individuals is “salt-sensitive”, exhibiting blood pressure increases or decreases with high or low salt consumption, respectively [5]. Mechanistic studies are beginning to shed light on the clinical presentation of salt sensitivity, including the influence of biological sex on disease manifestation. Large population studies indicate that salt-sensitive blood pressure responses are more pronounced in women, both in terms of the magnitude and prevalence of salt-induced blood pressure changes [6]. Despite these observations, numerous clinical and experimental studies suggest that renal mechanisms underlying salt-induced hypertension predominate in men and male animals [7]. In contrast, salt-sensitive hypertension has increasingly been associated with elevated aldosterone production in women and female rodent models. In females, this response may involve activation of the intra-adrenal renin-angiotensin system [8] and modulated by sex hormone receptor signaling [9, 10]. However, most studies investigating the mechanisms underlying salt-induced increases in blood pressure are conducted on young male rodents. Consequently, the long-term effects of excessive salt intake across the adult lifespan, including its coordinated impact on the heart and distinct vascular beds and how cardiovascular responses evolve in males and females during aging, remain incompletely understood.

To address this gap, we have recently conducted a long-term high-salt diet exposure (14 months) in male and female mice during aging [11]. The cardiovascular responses to salt markedly differed between the sexes. Females developed salt-sensitive hypertension associated with vascular dysfunction, including enhanced vascular contractility and increased arterial collagen deposition. In contrast, males developed cardiac and vascular remodeling, including left ventricular dilation, increased mass, and vascular dysfunction, without an increase in blood pressure. Subsequent dietary salt reduction normalized blood pressure in females and improved cardiac function in males but did not fully reverse structural cardiovascular and renal remodeling [11]. Thus, prolonged salt exposure produced persistent biological alterations despite recovery of major functional cardiovascular parameters.

Given the close integration of cardiovascular function with systemic metabolism, we hypothesized that these persistent effects of chronic HSD exposure would be accompanied by sustained alterations in the circulating metabolome. Specifically, based on metabolic processes implicated in cardiovascular remodeling and salt-induced stress, we expected alterations in lipid metabolism, energy metabolism, and amino acid homeostasis [12]. We further hypothesized that the metabolic response to chronic HSD exposure and its recovery following salt reduction would show different patterns in males and females, reflecting the distinct cardiovascular phenotypes observed in this cohort. To test these hypotheses, we analyzed plasma samples from the same cohort [11] using a targeted metabolite panel. Our primary objectives were to determine (i) which circulating metabolites and metabolic pathways are altered by long-term HSD exposure, (ii) to what extent these alterations normalize following prolonged dietary salt reduction, and (iii) whether the patterns of metabolic alteration and recovery differ between male and female mice. Finally, we explored associations between the identified metabolic alterations and the previously characterized cardiovascular phenotypes to assess whether systemic metabolic profiles reflect persistent cardiovascular remodeling.

## Methods

### Animals

All animal experiments were approved by the Malmö-Lund Committee for Animal Experiment Ethics (#7143/2018), conducted in accordance with European Directive 2010/63/EU, and reported following the ARRIVE guidelines (Animal Research: Reporting In Vivo Experiments, NC3Rs initiative, UK). Male and female C57BL/6JRj mice (20–24 weeks old) were obtained from Janvier Labs (Saint-Berthevin, France) and housed under controlled environmental conditions (12 h light-dark cycle, regulated temperature and humidity) in ventilated cages (Innovive, San Diego, CA USA) enriched with corn-cob bedding, nesting paper sheets, climbing ladders, cardboard cylinders, and wood blocks. Mice had access to food and water *ad libitum*.

Before any animal experimentation, mice were randomly assigned at the cage level to one of three 14-month dietary intervention groups using Research Randomizer (RRID: SCR_008563): normal diet (ND; 0.6% NaCl; RM3-P from Scanbur, Karlslunde, Denmark), high-salt diet (HSD; RM3-P with additional 8% NaCl), or a diet reversal group (REV), consisting of 7 months of HSD followed by 7 months of ND, for a total intervention period of 14 months (Supplementary Fig. 1A). Diets only differed with respect to salt content and were otherwise identical in caloric and macronutrient composition. Group sizes were as follows: ND *n* = 12 males, 15 females; HSD *n* = 13 males, 13 females; and REV *n* = 9 males, 10 females. Diet interventions were initiated at 9 months of age, after baseline characterization of the mice. Longitudinal arterial blood pressure measurements by tail-cuff plethysmography and longitudinal cardiac magnetic resonance imaging (MRI) in this mouse cohort were reported previously [11, 13].

### Plasma collection

Blood samples were collected by cardiac puncture under isoflurane anesthesia at the end of the intervention period. Whole blood was transferred into EDTA tubes and centrifuged at 1000 g for 10 min to obtain plasma, which was aliquoted and stored at -80 °C until further analysis.

### Metabolite profiling

Forty plasma samples were randomly selected for analysis because of analytical feasibility and resource considerations, resulting in the following group sizes: ND *n* = 5 males, 9 females; HSD *n* = 6 males, 7 females; and REV *n* = 5 males, 8 females. Sample selection was performed independently of cardiovascular phenotype, metabolomic outcome, or sample quality. Samples (20 µL) were vortex mixed with 20 µL MilliQ water and 160 µL methanol, and protein precipitation was facilitated by freezing at -20 °C for 3 h. Samples were then centrifuged, and the supernatant (160 µL) was collected and evaporated to dryness using a miVac Duo Concentrator (Genevac, Ipswich, UK) connected to a diaphragm vacuum pump (Vacuubrand, Wertheim, Germany). Then, 20 µL methoxyamine hydrochloride in pyridine was added (20 mg/mL), followed by incubation for 3 h at room temperature, and addition of 20 µL of *N*-methyl-*N*-trimethylsilyltrifluoroacetamide containing 1%(v/v) chlorotrimethylsilane, and incubation for another 30 min. A sample of bovine serum was derivatized and analyzed in parallel to ensure repeatability of the methodology. No internal standards were used, as the broad chemical diversity of the targeted metabolites precluded selection of a practical set of internal standards that adequately represented the chemical space covered by the method. Samples were analyzed on an Agilent 7890 A gas chromatograph coupled to an Agilent 5975 C VL MSD mass spectrometer equipped with a DB-5MS column (30 m x 250 μm x 0.25 μm, Agilent J&W GC Columns), as described previously [14]. Retention indexes were determined from analysis of a homologous series of *N*-alkanes. Metabolites were detected and identified against an in-house database derived from analysis of pure standards using a MATLAB-based script (MathWorks, Natick, MA-USA; RRID: SCR_001622) detailed elsewhere [15]. The targeted metabolite panel comprised 69 metabolites. Metabolites were retained for further analysis only if they met the following predefined quality criteria: retention index below 10 units deviation from database, signal-to-noise ratio over 10, and spectral match score obtained using NIST MS Search above 800 (https://chemdata.nist.gov).

### Statistical analysis

Metabolomics data were analyzed in MATLAB. After z-scoring, principal component analysis (PCA) was performed on the scaled data to explore global variance structure. Confidence ellipses (1 standard deviation) were computed based on the covariance of each group and plotted to visualize group dispersion. Supervised classification was performed using partial least squares discriminant analysis (PLS-DA) with treatment group encoded as a categorical variable. PLS models were fitted using the *plsregress* function with the optimal number of latent variables (LVs) determined using 10-fold cross-validation. For each model, the number of components minimizing the cross-validated mean squared error (MSE) of the response matrix was selected. Model performance was evaluated using cross-validated predictions. Classification accuracy was calculated as the proportion of correctly predicted samples. Confusion matrices were generated to assess class-specific performance. Predictive ability was evaluated using the cross-validated Q^2^ statistic, which estimates how well the model predicts new observations. Q^2^ was calculated from the predictive residual sum of squares (PRESS) obtained during cross-validation relative to the total sum of squares of the response matrix. Variable importance in projection (VIP) scores were calculated to identify metabolites contributing most strongly to group separation. Metabolites with VIP > 1 were considered important contributors to the model and were used for pathway analysis with MetaboAnalyst 6.0 (www.metaboanalyst.ca), using the Kyoto Encyclopedia of Genes and Genomes (KEGG) as pathway library [16].

To further characterize the metabolic shift induced by dietary reversal, binary PLS-DA models were constructed using only ND and HSD samples. Samples from the reversed diet (REV) group were excluded from model training and subsequently projected into the ND-HSD latent space using the PLS projection matrix, enabling evaluation of the position of REV samples along the ND-HSD axis without influencing model fitting.

GraphPad Prism 11 (San Diego, California; RRID: SCR_002798) was used for group comparisons. Data normality was assessed using Q-Q plots and the Shapiro-Wilk test. As no significant deviations from normality were detected, individual metabolites were analyzed by two-way ANOVA (for sex, diet and sex-diet interaction) followed by the Holm-Šídák’s multiple comparisons test. Differences were considered significant at a P-value threshold of 0.05.

Pearson correlation analyses were used to explore the relation between metabolite levels and PCA components with blood pressure and cardiac MRI parameters measured at 14 months of intervention in this mouse cohort [11, 13].

Data are presented as mean ± SD of *n* independent experiments (mice), unless otherwise stated.

## Results

Plasma samples for this metabolomics study were obtained from a cohort of mice previously characterized [11]. Briefly, mice were exposed to a high-salt diet from 9 to 23 months of age, followed in a subset by a seven-month return to a normal-salt diet. During periods of HSD exposure, estimated average salt intake was increased in the range of 18-20-fold in females and 20-23-fold in males, when compared to ND (Table 1). The associated cardiovascular phenotypes in males and females, and their partial reversibility have been reported previously and are summarized in Supplementary Figure S1B-M and Supplementary Table S1.

**Table 1:** Estimated food intake and NaCl consumption (g/mouse/week) estimated from gross food intake for the mice in normal diet (ND), high-salt diet (HSD), and reversed diet (REV) groups

	Female			Male		
	ND (*n* = 3)	HSD (*n* = 3)	REV (*n* = 3)	ND (*n* = 3)	HSD (*n* = 6)	REV (*n* = 6)
**Food intake**						
baseline	19.1 ± 2.7	22.6 ± 4.2	21.6 ± 2.5	26.7 ± 3.1	29.3 ± 3.5	28 ± 3.6
First month of diet intervention	21.7 ± 2.9	27.5 ± 2.1	28.3 ± 1.4	26.5 ± 2.4	39.6 ± 5.8	44.6 ± 8.9
After diet reversal	24 ± 2.6	30.3 ± 5.6	23.4 ± 1.5	25.1 ± 3.9	43.8 ± 5.5	32.3 ± 2.6
**NaCl consumption**						
baseline	0.11 ± 0.02	0.14 ± 0.03	0.13 ± 0.02	0.16 ± 0.02	0.18 ± 0.02	0.17 ± 0.02
First month of diet intervention	0.13 ± 0.02	2.37 ± 0.18	2.43 ± 0.12	0.16 ± 0.02	3.40 ± 0.50	3.83 ± 0.77
After diet reversal	0.14 ± 0.02	2.61 ± 0.48	0.14 ± 0.01	0.15 ± 0.02	3.77 ± 0.47	0.19 ± 0.02

Cage-average food intake was measured at baseline, during the first month of diet intervention, and during the 2 months that followed the diet reversal. Data is mean ± SD of 3 or 6 cages, as indicated in parenthesis

To determine whether these cardiovascular phenotypes were accompanied by systemic metabolic alterations, we performed targeted plasma metabolomics, including glycolytic and Krebs cycle intermediates, fatty acids, sugars, amino acids and related intermediates. Of the 69 targeted metabolites, 48 met the predefined quality criteria for spectral identification and retention index matching and were detected in all analyzed samples [14].

**Fig. 1:**
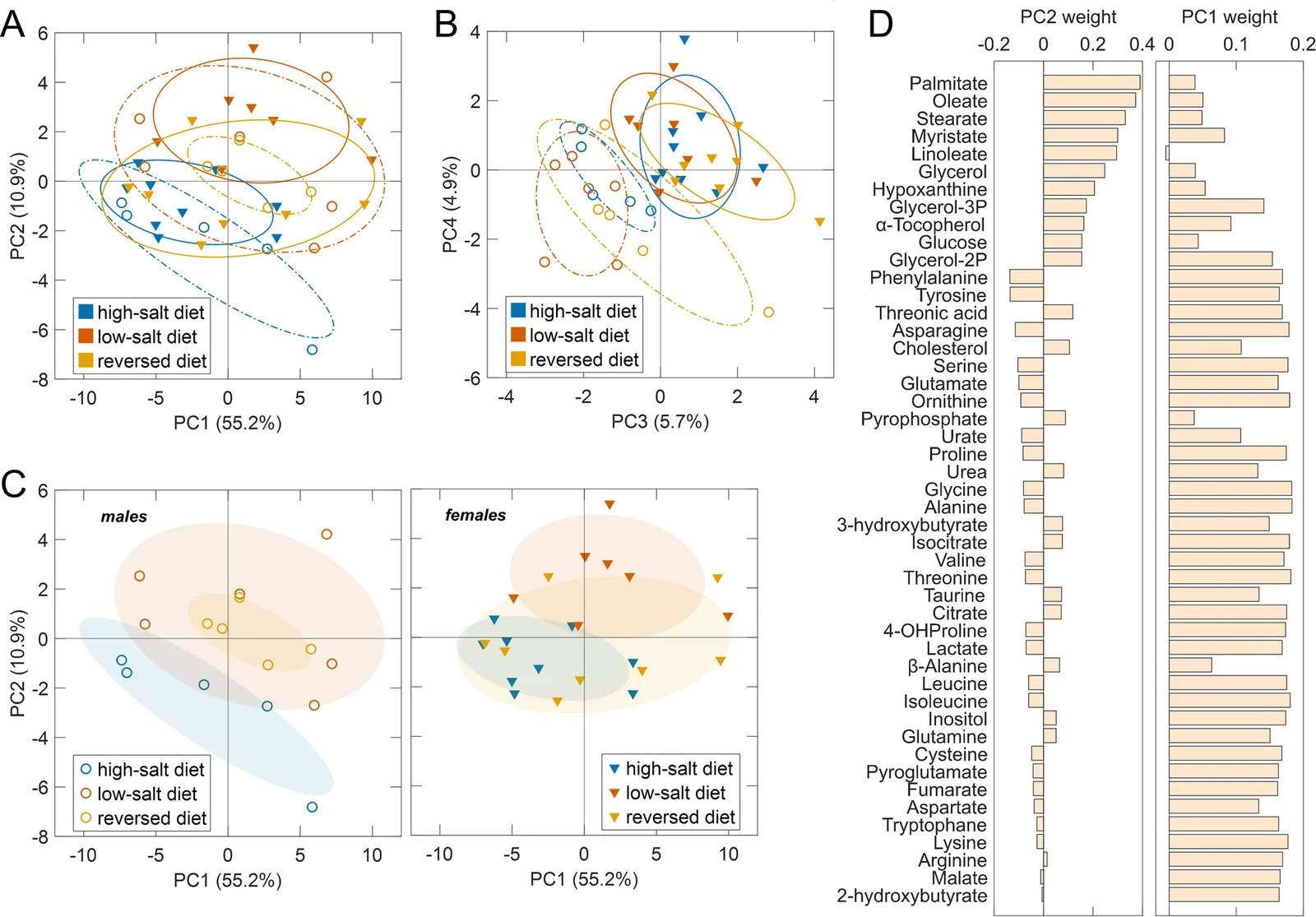
Principal component analysis (PCA) of Z-scored relative metabolite levels. (**A**) PCA score plot showing the effect of HSD exposure on the metabolite profile along principal component 2 (PC2). (**B**) PC3 contains information on sex differences in metabolite levels, with PC3 and PC4 providing full separation of male (circles) and female (triangles) mice. (**C**) Visualization of males and females separately, indicating limited metabolic recovery in female mice and near-complete recovery in male mice in the reversed diet group. (**D**) PC1 and PC2 for each metabolite, ordered by the absolute value of the PC2 loading. Ellipsoids in score plots represent SD of the respective treatment-by-sex group (dashed lines=males; solid lines=females)

PCA was first used to explore global metabolic differences across experimental groups. The PCA score plot revealed a partial separation of HSD and ND groups along principal component 2 (PC2), accounting for 10.9% of the variance in the data (Fig. 1A). Separation between male and female mice was observed in later components (Fig. 1B), suggesting distinct metabolic profiles between sexes. When the sexes were analyzed individually, REV animals clustered closely with ND animals for male mice, indicating a reinstatement of normal metabolic control (Fig. 1C). In contrast, in female mice, REV largely overlapped with the HSD group, consistent with incomplete metabolic normalization following dietary reversal (Fig. 1C). The metabolites contributing most strongly to salt-induced metabolic regulation included several fatty acids (Fig. 1D).

Given these different patterns observed in males and females, we next performed supervised multivariate analyses separately in male and female mice. PLS-DA models were constructed independently for each sex to identify metabolites associated with HSD exposure and withdrawal. In males, the optimal model yielded a coefficient of determination (R^2^) of 0.667 and a cross-validated predictive ability statistic (Q^2^) of -0.520, indicating poor predictive performance and overfitting (Supplementary Figure S2).

In females, the PLS-DA model showed improved performance with R^2^ = 0.763 and Q^2^ = 0.449. Score plots suggested that female REV mice did not fully revert to the ND profile and appeared distinct from both ND and HSD groups (Supplementary Figure S3), indicating persistent metabolic alterations after reversal to a normal-salt diet.

**Fig. 2:**
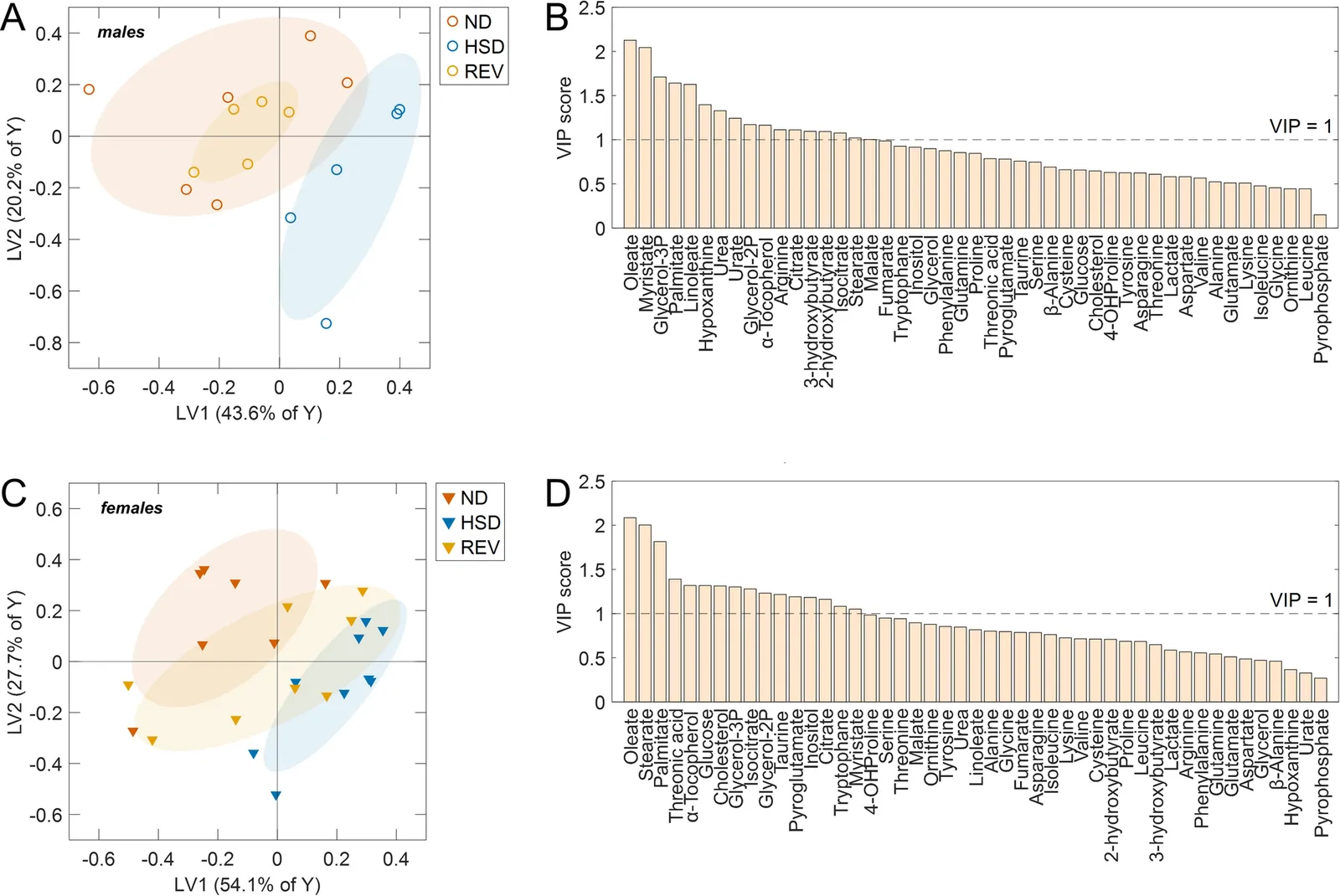
Partial least squares discriminant analysis (PLS-DA) of normal diet (ND) and high-salt diet (HSD)-fed male (**A-B**) and female (**C-D**) mice using 2 latent variables (LVs). PLS-DA score plots show separation between ND and HSD clusters in latent space, which is especially clear for females. The reversed diet (REV) group was not used to construct the PLS model but was projected into the LV space (**A/C**). Variable importance in projection (VIP) scores for each metabolite are shown for the PLS-DA models of male (**B**) and female (**D**) mice. Symbols and ellipsoids in the score plots represent individual mice and the respective group SD

To further assess metabolic recovery, binary PLS-DA models were constructed using ND and HSD groups only and were then used to classify REV mice by *post hoc* projection into the ND-HSD latent space. In both sexes, two latent variables were optimal. In males, the PLS-DA model (R^2^ = 0.638, Q^2^ = 0.144) provided moderate separation of ND and HSD groups, and all REV mice were classified as ND based on the relative distances to ND and HSD centroids (Fig. 2A), supporting a near-complete metabolic recovery. The top five metabolites contributing to group discrimination based on VIP scores were oleate, myristate, palmitate, linoleate and urea (Fig. 2B). In females, the PLS-DA model demonstrated higher predictive performance based on the model validation metrics (R^2^ = 0.818, Q^2^ = 0.675) and fully separated ND and HSD mice. However, only three of the eight REV mice were classified as ND (Fig. 2C), indicating incomplete recovery in most animals. The top five contributing metabolites were oleate, stearate, palmitate, threonic acid and glucose (Fig. 2D), which were also identified as significantly altered by HSD in univariate analyses (Supplementary Figure S4).

From these PLS-DA models, metabolites with VIP > 1 were used for pathway analysis (Supplementary Figure S5). Pathways considered to be most relevant (*P* < 0.05 and hits > 1) in HSD versus ND that were common to males and females included biosynthesis of unsaturated fatty acids, citrate cycle, and glyoxylate and dicarboxylate metabolism. In addition, in males, HSD impacted arginine biosynthesis, purine metabolism, and alanine, aspartate and glutamate metabolism. Galactose metabolism was exclusively modified by HSD in females.

**Fig. 3:**
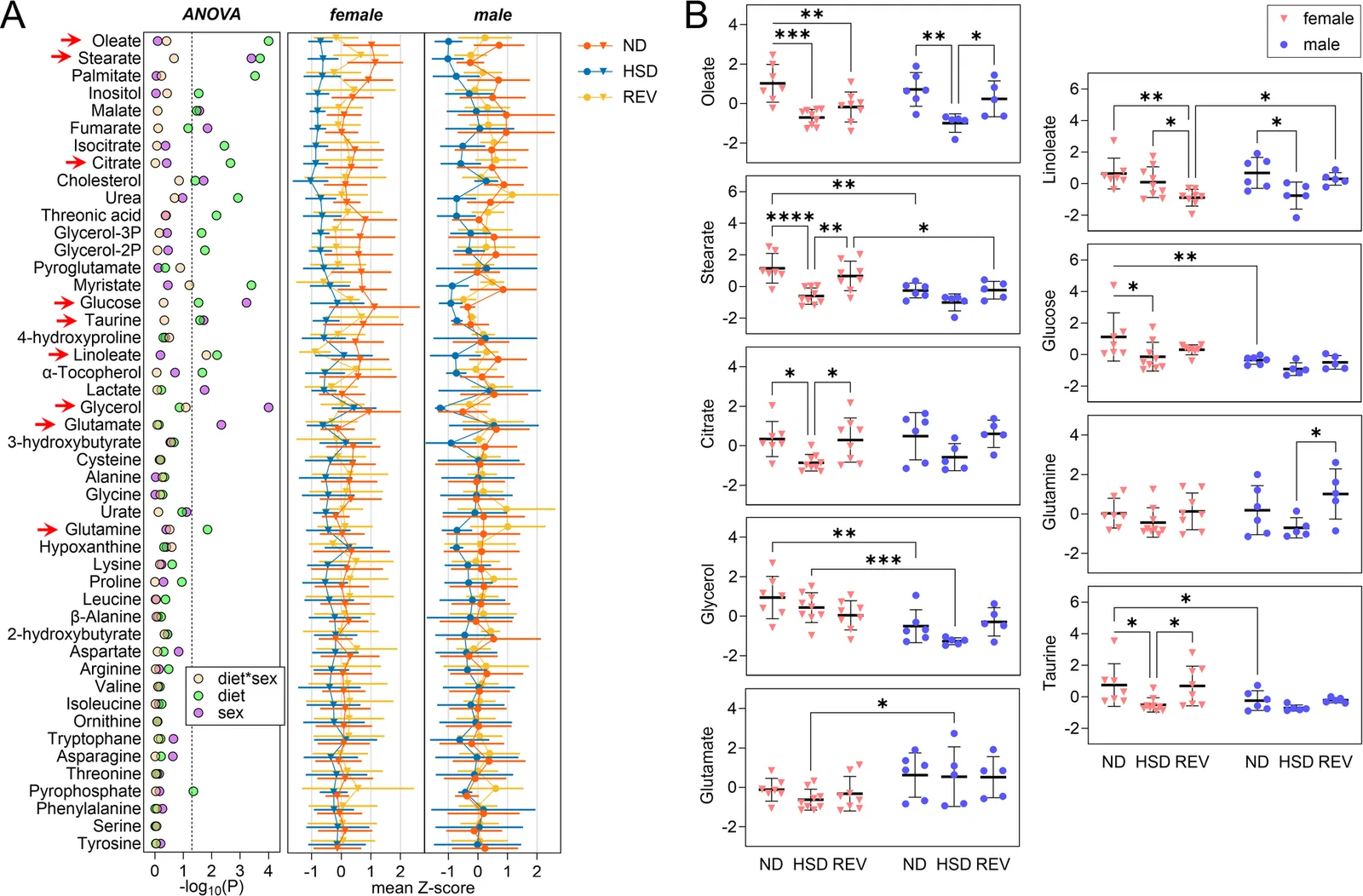
Relative differences in levels of individual metabolites. (**A**) P-values from ANOVA and relative Z-score for normal diet (ND), high-salt diet (HSD) and reversed diet (REV)-fed mice. (**B**) Selected metabolites demonstrating representative differences across groups, with y-axis representing the standardized relative abundance (Z-score). Red arrows in (**A**) indicate the metabolites plotted in (**B**). Data points represent each mouse, overlaid onto the respective mean ± SD. Triangles and dots represent female and male mice, respectively. Significant differences from Holm-Šídák’s multiple comparisons tests, following significant ANOVA effects, are indicated as follows: *P < 0.05, **P < 0.01, ***P < 0.001, ****P < 0.0001

To identify specific metabolite changes associated with diet and sex, we performed two-way ANOVA. A total of 19 metabolites were significantly affected by diet, while 9 were influenced by sex (Fig. 3A; Supplementary Table S2). Five metabolites (malate, cholesterol, taurine, glucose, and stearate) showed significant effects of diet and sex without significant diet × sex interaction. A significant diet × sex interaction was observed only for linoleate (interaction *P* = 0.0155, diet *P* = 0.0065, sex *P* = 0.644), indicating a different response to dietary intervention in male and female mice. In males, linoleate levels decreased under HSD and returned to ND levels in the REV group. In contrast, linoleate levels were not altered by HSD in females but were reduced in the REV group (Fig. 3B). The low number of interactions between sex and diet is likely attributable to the small number of animals in each group, which limited the statistical power of these analyses.

**Fig. 4:**
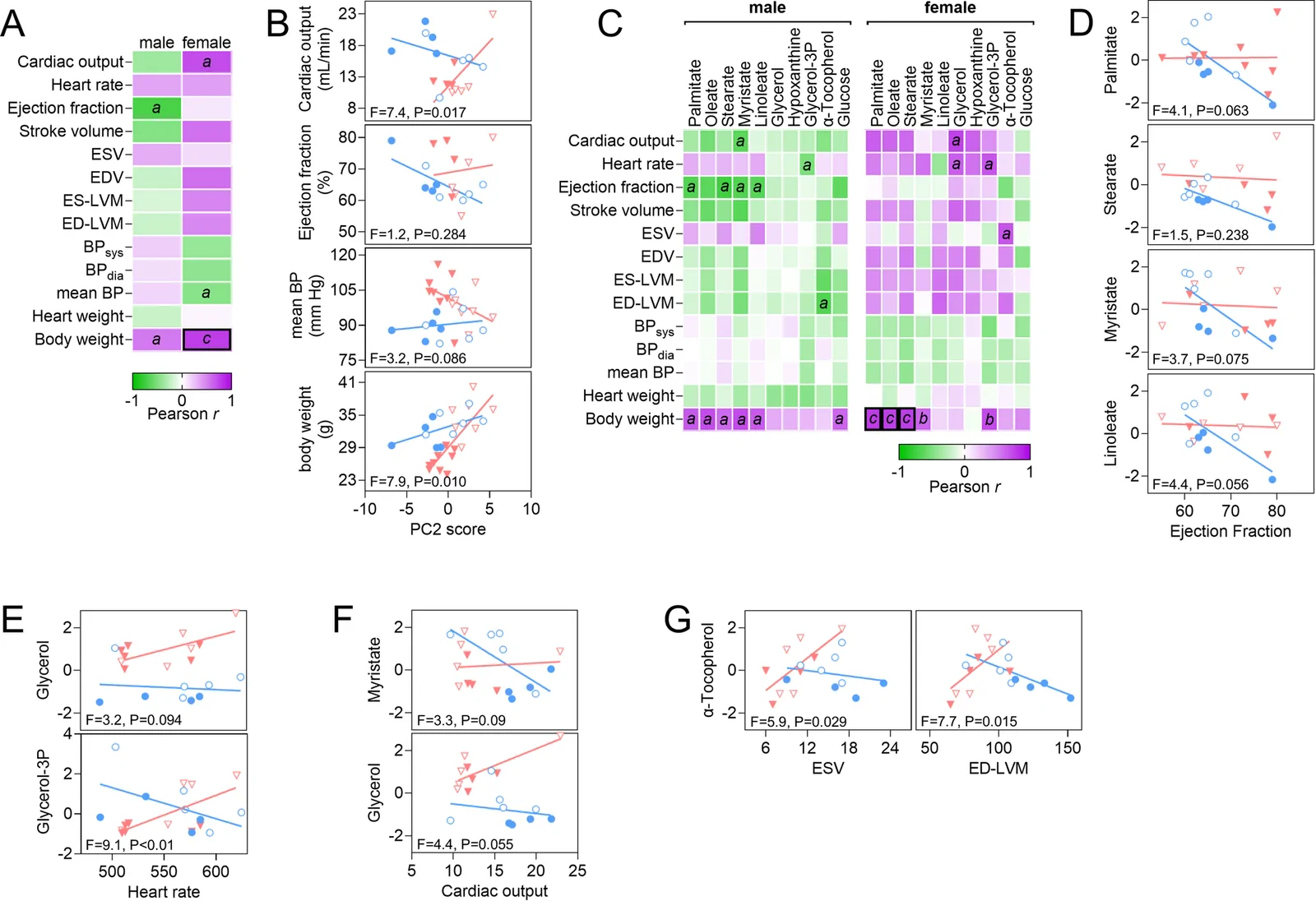
(**A**) Correlation between principal component analysis PC2 score and cardiovascular parameters previously measured in male and female mice fed a normal diet (ND) or high-salt diet (HSD) throughout the intervention period. (**B**) Representation of significant correlations between PC2 scores and cardiac output, ejection fraction, mean blood pressure (BP) and body weight in male (circles) and female (triangles) mice. (**C**) Pearson’s *r* values for correlations between the top ten metabolites contributing to PC2 and cardiovascular variables. (**D-G**) Relationship between selected metabolites, represented as Z-score, and ejection fraction, cardiac output or heart rate in male and female mice. Open and filled symbols represent ND and HSD, respectively. Symbols indicate significance based on unadjusted P-values: ^*a*^*P* < 0.05, ^*b*^*P* < 0.01, ^*c*^*P* < 0.001. Correlations shown in boxes survived adjustment for multiple testing (P_adjusted_ < 0.05). Exact P-values and adjustment for multiple testing are shown in Supplementary table S3. Lines indicate linear regression fits. F and P values in panels B and D-G result from a sum-of-squares F test to compare whether the slope is significantly different between males and females. Abbreviations: ESV, end-systolic volume; EDV, end-diastolic volume; ES-LVM, end-systolic left ventricle mass; ED-LVM, end-diastolic left ventricle mass; BP, blood pressure; BP_sys_, systolic blood pressure; BP_dia_, diastolic blood pressure

To link metabolic changes with cardiovascular phenotypes, we correlated PCA-derived scores with functional parameters (Fig. 4A-B; Supplementary Table S3). For this analysis, we selected the PC that captured diet-related variation, PC2, and correlated its scores with functional endpoints (see Fig. 1). In females, PC2 scores correlated positively with cardiac output (*r* = 0.714, *P* = 0.015) and negatively with mean arterial blood pressure (*r* = -0.460, *P* = 0.042). In males, PC2 scores correlated negatively with ejection fraction (*r* = -0.716, *P* = 0.015). In both sexes, PC2 scores were positively correlated with body weight (females: *r* = 0.779, *P* = 0.0002; males: *r* = 0.533, *P* = 0.046). After adjustment for multiple correlations in this exploratory analysis, only body weight in females was significantly correlated with PC2 (P_adjusted_ = 0.0026). Sex differences were significant for the relationship between PC2 and cardiac output and body weight (Fig. 4B).

Analysis of the top ten metabolites contributing to PC2 revealed significant correlations between cardiovascular parameters and several fatty acids, glycerol and glycerol-3-phosphate (Fig. 4C-G; Supplementary Table S3). Notably, α-tocopherol showed different associations in males and females, correlating negatively with end-diastolic left ventricular mass in males and positively with end-systolic volume in females (Fig. 4C-G). Similar, albeit weaker, relationships were observed when all diet groups were analyzed together (Supplementary Figure S6). Only body weight in females was significantly correlated with the fatty acids palmitate (P_adjusted_ = 0.0358), oleate (P_adjusted_ = 0.0208) and stearate (P_adjusted_ = 0.0244) after adjustment for multiple correlations (Supplementary Table S3). Furthermore, significant sex differences were observed for correlations between glycerol-3-phosphate and heart rate (Fig. 4E), as well as α-tocopherol and both end-systolic volume and end-diastolic left ventriclular mass (Fig. 4G).

Overall, these exploratory findings demonstrate different metabolic responses and recovery trajectories in male and female mice, with fatty acids emerging as key contributors and metabolite-phenotype relationships for glycerol-3-phosphate and α-tocopherol displaying opposing patterns between males and females.

## Discussion

This study demonstrates that chronic high-salt intake is associated with alterations in the circulating metabolome of aging mice and that the patterns of metabolic response and recovery differ between males and females. These metabolic changes were only partially aligned with the previously characterized cardiovascular phenotypes. Most notably, dietary salt reduction was accompanied by normalization of blood pressure in females, whereas the plasma metabolite profile remained different from that of ND animals. Together with the different metabolic patterns observed in males, these findings indicate that cardiovascular and metabolic responses to prolonged salt exposure do not necessarily develop or resolve in parallel.

A central insight from our metabolomic analyses is that the metabolic response to HSD was characterized by coordinated changes of modest magnitude across several metabolite classes rather than large alterations in a small number of individual metabolites. Metabolites affected by diet included glucose, glycerol and glycerol-3-phosphate, fatty acids, cholesterol, and taurine, pointing towards alterations in processes related to energy and lipid metabolism relevant to cardiometabolic disease [17]. This interpretation was further supported by pathway analysis, which identified biosynthesis of unsaturated fatty acids, the citrate cycle, and glyoxylate and dicarboxylate metabolism among pathways affected by HSD in both sexes. For example, glycerol-3-phosphate represents an important intersection between glucose and lipid metabolism and participates in cellular redox metabolism through the glycerol-3-phosphate shuttle, which transfers cytosolic reducing equivalents to the mitochondrial respiratory chain [18, 19]. Its potential relevance to cardiometabolic disease is supported by experimental evidence that glycerol-3-phosphate can be released by the injured kidney [20] and that disturbances in glycerol-3-phosphate phosphatase activity affect glucose and lipid metabolism under metabolic stress [21].

Taurine was reduced by HSD and contributed to the discrimination of dietary groups in female mice. This finding is consistent with reports of altered circulating taurine levels in hypertension [22, 23] and with reduced plasma taurine during HSD exposure in rats [24]. Although these observations do not establish a causal role, they raise the possibility that reduced taurine availability contributes to female-specific blood pressure response to chronic salt exposure. This interpretation is supported by evidence linking higher dietary taurine intake to cardiovascular benefits [25] and by studies suggesting that taurine supplementation may lower blood pressure [26, 27] and exert protective effects across a range of cardiovascular and metabolic conditions (e.g., [28, 29]).

Fatty acids emerged as prominent contributors to HSD-associated metabolic variation in both male and female mice. This observation is consistent with evidence that excessive salt intake can alter hepatic lipid metabolism and systemic lipid handling [30]. Fatty acids are also central for myocardial energy metabolism and participate in vascular regulation [31, 32], providing a potential biological context for the observed associations between circulating fatty acids and cardiac structural and functional parameters.

Among these metabolites, linoleate was particularly notable because it was the only metabolite showing a statistically significant diet × sex interaction. Linoleate is an essential omega-6 polyunsaturated fatty acid involved in endothelial function, vascular tone regulation, and cardiometabolic health [33–35]. In males, linoleate decreased during continuous HSD exposure and was comparable to ND following dietary reversal, whereas in females the pattern differed, with reduced levels particularly evident after dietary reversal. Because linoleate is an essential dietary fatty acid, circulating concentrations may reflect substrate availability as well as endogenous lipid handling. Food-consumption measurements available during the first month of intervention and following dietary reversal did not indicate reduced food intake during HSD exposure, arguing against reduced overall food consumption as a straightforward explanation. Nevertheless, food intake was not continuously measured throughout the intervention, and effects on nutrient absorption or lipid handling cannot be distinguished by the present study. Although the remaining metabolite changes cannot be considered formally sex-specific responses to HSD, their occurrence alongside distinct cardiovascular phenotypes in males and females raises the possibility that similar metabolic alterations may accompany different physiological adaptations depending on biological sex [36–38].

Pathway analysis provided additional evidence that HSD-associated metabolic patterns were not identical in males and females. Biosynthesis of unsaturated fatty acids, the citrate cycle, and glyoxylate and dicarboxylate metabolism were identified in both sexes, whereas arginine biosynthesis, purine metabolism, and alanine, aspartate and glutamate metabolism were identified in males, and galactose metabolism in females. Arginine availability has been associated with cardiovascular outcomes [39], while alterations in purine metabolism occur during metabolic stress states associated with obesity and cardiovascular disease [40]. Galactose metabolism is likewise linked to cellular and cardiovascular metabolic processes [41]. These findings suggest that prolonged HSD exposure affects several aspects of systemic metabolism and provide a pathway-level context for the individual metabolite changes.

Importantly, the metabolic alterations observed in response to HSD were associated with the cardiovascular phenotypes previously characterized in this cohort. Diet-associated variation in the metabolomic profile, as well as several individual metabolites, correlated predominantly with measures of cardiac structure and function, whereas associations with arterial blood pressure were less prominent. These relationships further support a connection between systemic metabolic alterations and cardiovascular remodeling during chronic salt exposure and suggest that the circulating metabolome may reflect aspects of the cardiovascular phenotype that are not captured by blood pressure alone.

A distinctive feature of the present study is the prolonged duration of HSD exposure and the inclusion of a diet reversal group. Preclinical studies commonly use diets containing 4–8% NaCl over considerably shorter periods to induce cardiovascular changes [42–45]. In contrast, our 14-month intervention allowed us to examine the consequences of sustained salt exposure [5] across a substantial proportion of the murine lifespan and, through the REV group, the metabolic state after seven months of HSD exposure followed by an equally long period of salt reduction. This study design not only resembles a scenario in which dietary modification is initiated in middle age after prolonged exposure to excess salt but also introduces an important temporal dimension to the interpretation of the observed metabolic phenotypes.

The differences between REV males and females may therefore reflect more than different capacities for metabolic recovery. In males, the REV metabolomic profile was closer to ND, whereas females retained a profile distinct from ND and, for several features, also from continuous HSD consumption. One interpretation is that HSD-induced metabolic alterations are more readily reversible in males than in females. However, an equally relevant possibility is that the metabolic responses develop with different kinetics. If alterations become established earlier during HSD exposure in females, seven months of HSD may be sufficient to induce a metabolic state that persists despite subsequent salt reduction. Conversely, some of the alterations observed after 14 months of continuous HSD in males may develop more gradually, such that withdrawal of HSD after seven months occurs before the metabolic phenotype is fully established. The REV group could therefore capture differences in both the development and persistence of HSD-associated metabolic alterations. This possibility is particularly interesting considering the distinct cardiovascular trajectories previously observed in this cohort, with females developing salt-sensitive hypertension and males predominantly developing progressive cardiac and vascular remodeling without increased blood pressure [11].

This temporal interpretation also provides a broader context for the apparent dissociation between metabolic and cardiovascular recovery. In females, blood pressure normalized following salt reduction while the metabolomic profile remained distinct from ND, consistent with the persistent vascular remodeling previously observed in this model [11]. The female REV phenotype may therefore represent an alternative metabolic state following prolonged salt exposure and withdrawal that is distinct from both the continuously exposed and control conditions. In males, REV animals shifted towards the ND metabolic profile in parallel with improvement in cardiac function. Collectively, the findings suggest that metabolic and cardiovascular adaptations to chronic salt exposure have different temporal dynamics and that normalization of an overt physiological parameter does not necessarily indicate reversal of the broader biological response.

The present study has several limitations. The targeted metabolomics approach captured only a predefined subset of circulating metabolites and may therefore have missed additional metabolic pathways affected by chronic salt exposure and dietary reversal. Moreover, plasma provides an integrated systemic readout but does not identify the tissue sources of the observed metabolic alterations. This study has not acquired metabolomic data in a longitudinal manner and, therefore, misses the trajectory of metabolic recovery and a possible age-diet interaction. The associations between individual metabolites and cardiovascular phenotypes are correlative and do not establish causality or a mechanistic link. The relatively small sample size limits statistical power, particularly for sex-stratified analyses, and most of these associations did not remain statistically significant after FDR correction. Therefore, these metabolite-cardiovascular associations should be interpreted as exploratory findings requiring validation in an independent cohort. An additional consideration is that HSD-fed mice showed reduced body weight, whereas higher sodium intake in humans has been associated with greater body weight and obesity-related outcomes in observational studies [46, 47]. Available measurements did not indicate reduced food consumption during HSD exposure, suggesting that reduced caloric intake alone is unlikely to explain the lower body weight. Altered nutrient absorption, energy metabolism, fluid balance, or other model-specific responses may nevertheless contribute to both body weight and metabolomic changes.

In conclusion, chronic HSD exposure in aging mice was associated with alterations across several components of the circulating metabolome, particularly metabolites related to lipid and energy metabolism. Male and female mice showed different metabolic patterns during continuous HSD exposure and following dietary salt reduction, although a formal diet × sex interaction at the individual-metabolite level was demonstrated only for linoleate. Importantly, the REV groups suggest that these differences may involve not only the extent of metabolic recovery but also differences in the timing with which HSD-associated metabolic alterations become established and persist after salt withdrawal, potentially resulting in metabolic states distinct from those observed under continuous HSD or ND. Together with our previous cardiovascular findings [11], the present data indicate that metabolic and cardiovascular adaptations to chronic salt exposure can follow different trajectories and that recovery of blood pressure or cardiac function does not necessarily signify normalization of the broader systemic response. Longitudinal studies will be important to define these trajectories and determine the mechanisms underlying their differences between males and females.

## Supplementary Information

Below is the link to the electronic supplementary material.

Supplementary Material 1

Supplementary Material 2

Supplementary Material 3

Supplementary Material 4

## Data Availability

The authors confirm that the data supporting the findings of this study are available within the article and its supplementary materials.
