# Supplementary material for: Persistent metabolic remodeling after dietary salt reduction reveals sex differences in the adaptation to chronic high-salt intake
Source: Biol Sex Differ. 2026 Nov 12;17:180. doi: 10.1186/s13293-026-01017-1 (PMC13760752; doi:10.1186/s13293-026-01017-1)

Supplementary material for Göktaş *et al.*

"Persistent metabolic remodeling after dietary salt reduction reveals sex-dependent adaptation to chronic high-salt intake"

Supplementary Table S1. Results from 2-way ANOVA in Figure 1. Abbreviations: SS, sum of squares; DF, degrees of freedom; MS, mean square; DFn, DF for numerator (effect); DFd for denominator (error/residuals). Green shade indicates P<0.05, with darker color representing higher significance.

Supplementary Table S2. Results from 2-way ANOVA in Figure 3. Abbreviations: SS, sum of squares; DF, degrees of freedom; MS, mean square; DFn, DF for numerator (effect); DFd for denominator (error/residuals). Green shade indicates P<0.05, with darker color representing higher significance. #TMS indicates the number of trimethylsilyl (TMS) groups present in the derivatized metabolite.

Supplementary Table S3. Results from correlation analysis in Figure 4 including Pearson r, raw P-values, and false discovery rate (FDR)-adjusted P-values. Whenever a correlation was significant for male and/or female mice, slopes of linear fits were compared between sexes using a sum-of-squares F test for each parameter.

Supplementary Figure S1. Sex-specific cardiovascular effects of chronic high-salt intake and impact of diet reversal. (A) Schematic summary of the study design with interventions with normal diet (ND) and high-salt diet (HSD) during adulthood and aging. (B) Body weight at the end of the study. (C) Systolic, diastolic and mean arterial blood pressure (BP) depicting salt-sensitive hypertension in HSD females compared to REV. Stroke volume (D), end-diastolic volume (E), end-systolic volume (F), end-diastolic left ventricular mass (G), and end-systolic left ventricular mass (H) indicating cardiac remodeling and structural changes in males. (I) Heart rate in beats per minute (bpm). (J) Cardiac output showing HSD-induced functional remodeling in males. (K) Preserved ejection fraction in both sexes. (L) HSD induced negligible within-sex variations in heart weight, but (M) large differences in heart weight-to-body weight ratio. Data is shown as datapoints representing each mouse overlaid onto the respective mean ± SD. Triangles and dots represent female and male mice respectively. Significant differences from Holm-Šídák's multiple comparisons tests (after significant ANOVA effects) are indicated as follows: *P<0.05, **P <0.01, ***P<0.001, ****P<0.0001. This data has been previously reported by Duarte *et al*. (reference 11).

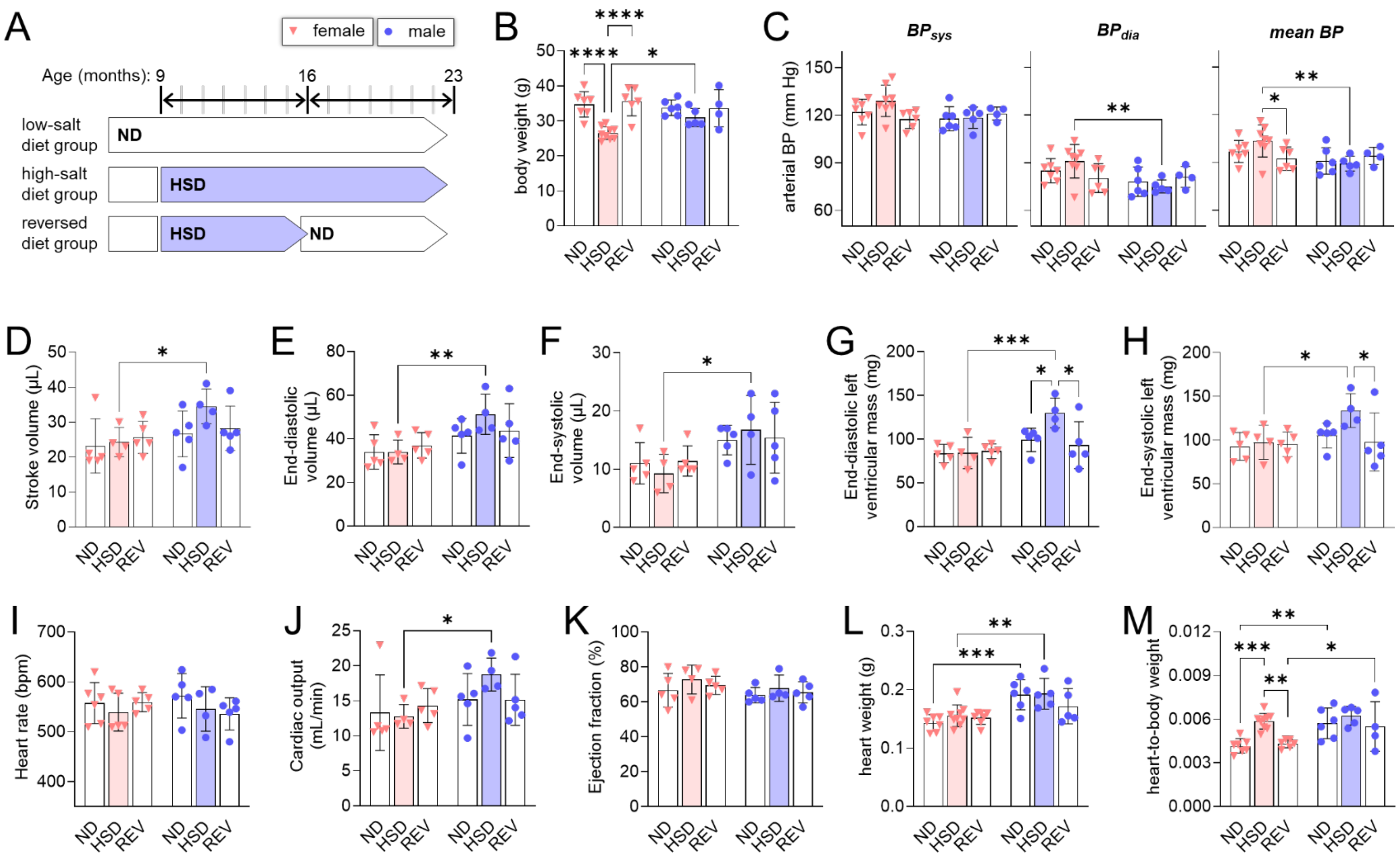

Supplementary Figure S2. Partial least squares discriminant analysis (PLS-DA) for male mice using 3 latent variables (LVs) that account for 50.9% of the variance explained. (A-B) Visualization of the distribution of samples in the latent space in score plots of LV1 *versus* LV2 (A) and LV2 *versus* LV3 (B). Each point represents an individual mouse in high-salt (blue), low-salt (orange) and reversed (yellow) diet groups. Ellipsoids represent SD of the respective group. (C) VIP score for each metabolite. The dashed line represents VIP=1.

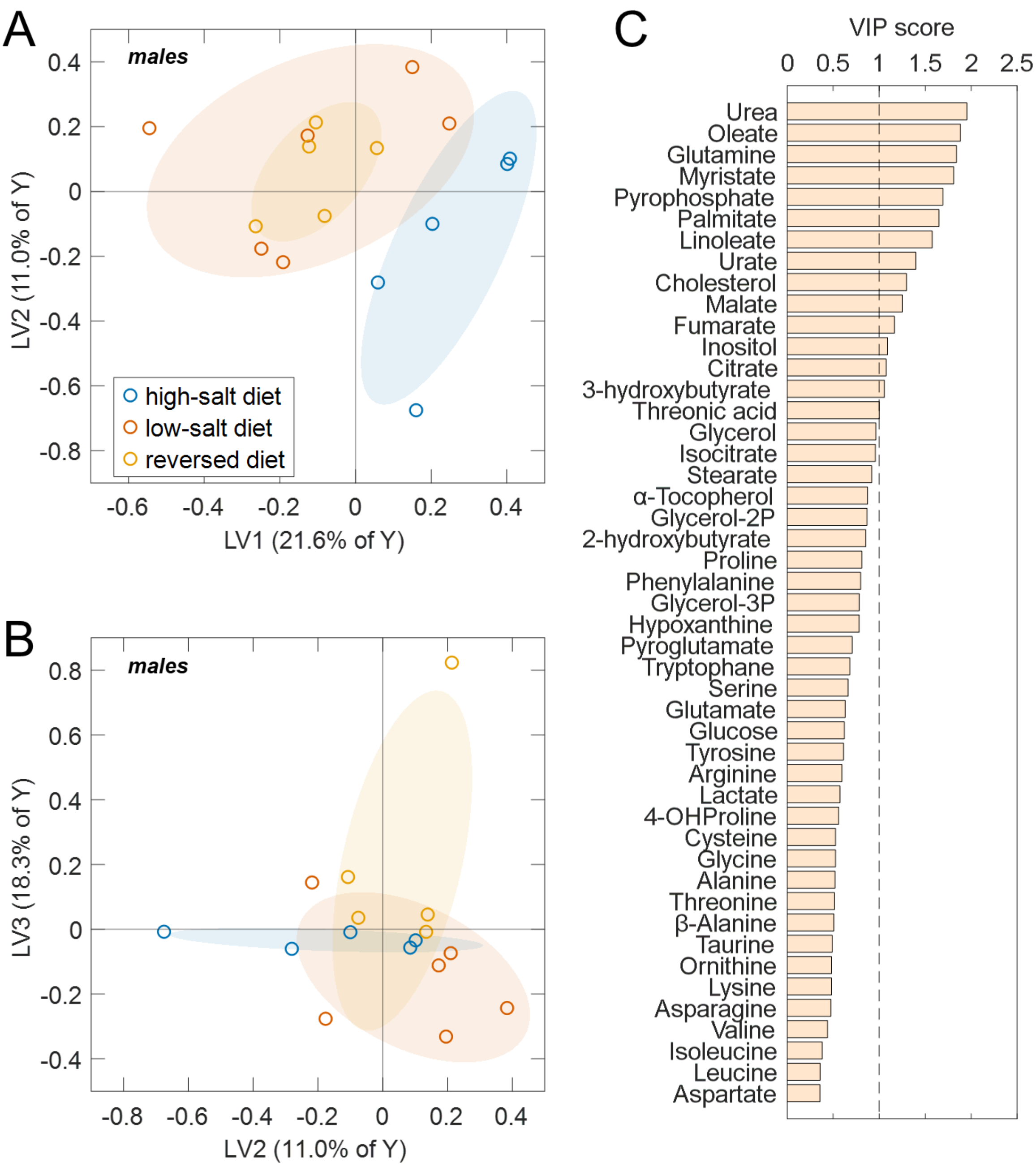

Supplementary Figure S3. Partial least squares discriminant analysis (PLS-DA) for female mice using 4 latent variables (LVs) that account for 74.4% of the variance explained. (A-B) Visualization of the distribution of samples in the latent space in score plots of LV1 *versus* LV2 (A) and LV3 *versus* LV4 (B). Each point represents an individual mouse in high-salt (blue), low-salt (orange) and reversed (yellow) diet groups. Ellipsoids represent SD of the respective group. (C) VIP score for each metabolite. The dashed line represents VIP=1.

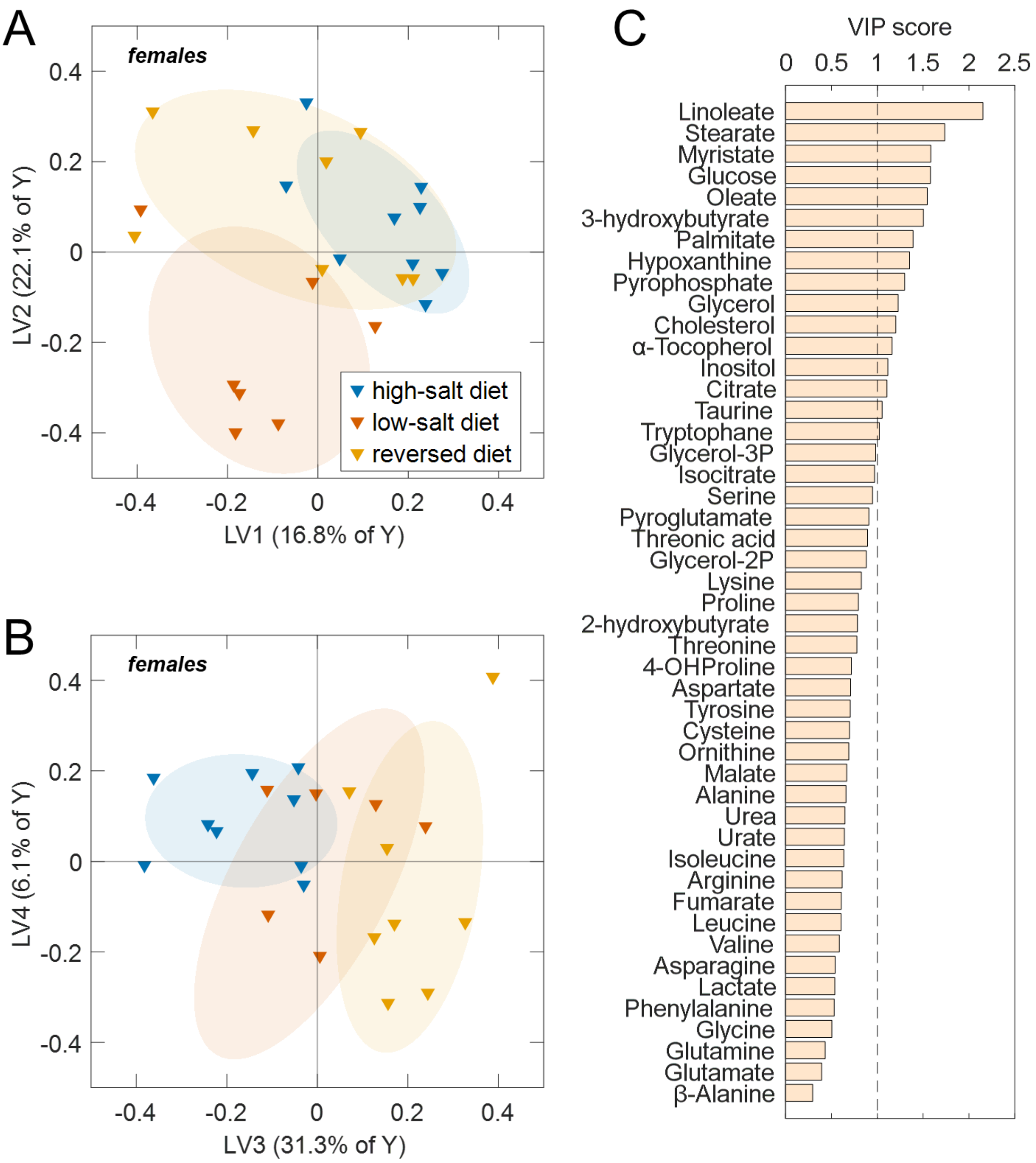

Supplementary Figure S4. Volcano plots of high-salt diet-induced changes on metabolite levels (HSD *versus* ND) in male (A) and female (B) mice. X-axis displays the fold-change, Y-axis displays P-values resulting from 2-tailed Student t-tests. The horizontal dashed line represents P=0.05.

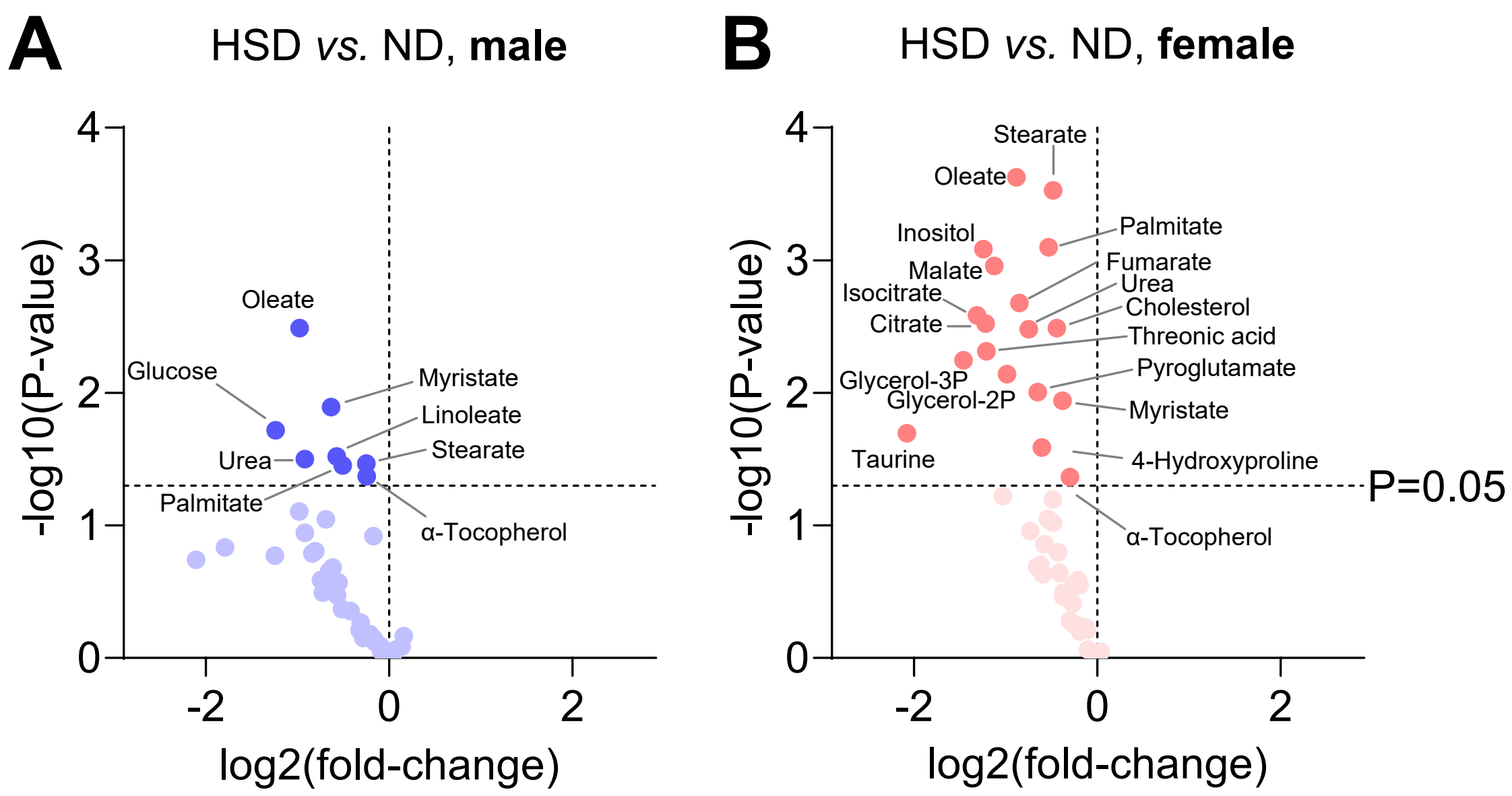

Supplementary figure S5. Metabolic pathway analysis on metabolites with VIP>1 in PLS-DA for ND *versus* HSD in females (A) and males (B). Graphs show uncorrected P-values and number of hits (*i.e.*, metabolites identified) in the respective pathway. Only pathways with P>0.1 are displayed. Bars highlighted with a back border represent pathways which P-value survived false discovery rate correction. Dashed line indicates P=0.05.

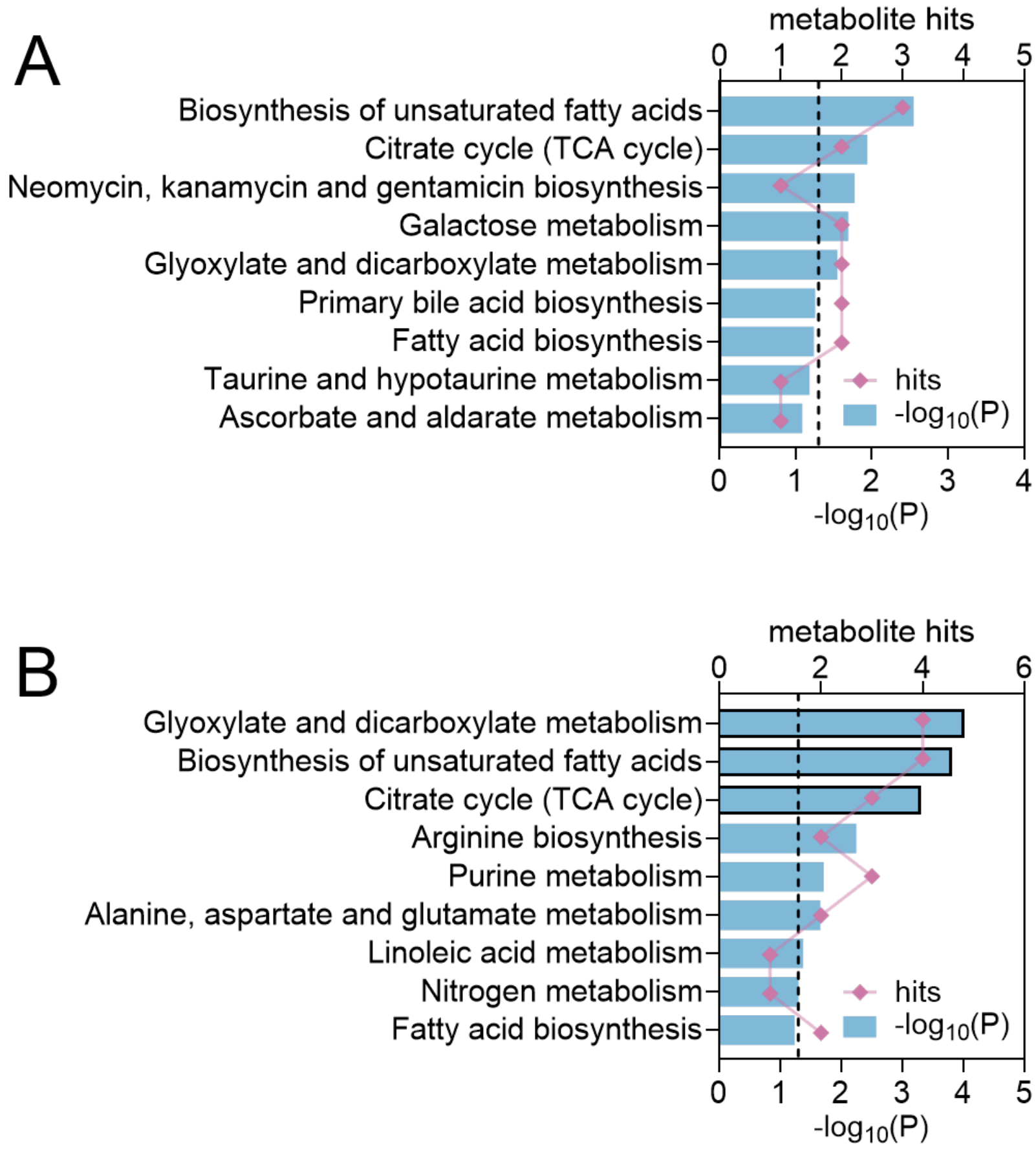

Supplementary Figure S6. Correlation analysis of PC2 score (A) and top 10 metabolites in PC2 (B) with cardiovascular parameters previously measured in male and female mice from the 3 diet intervention groups (all treatments). Letters indicate significance: [a] $P<0.05$, [b] $P<0.01$, [c] $P<0.001$. Abbreviations: ESV, end-systolic volume; EDV, end-diastolic volume; ES-LVM, end-systolic left ventricular mass; ED-LVM, end-diastolic left ventricular mass.

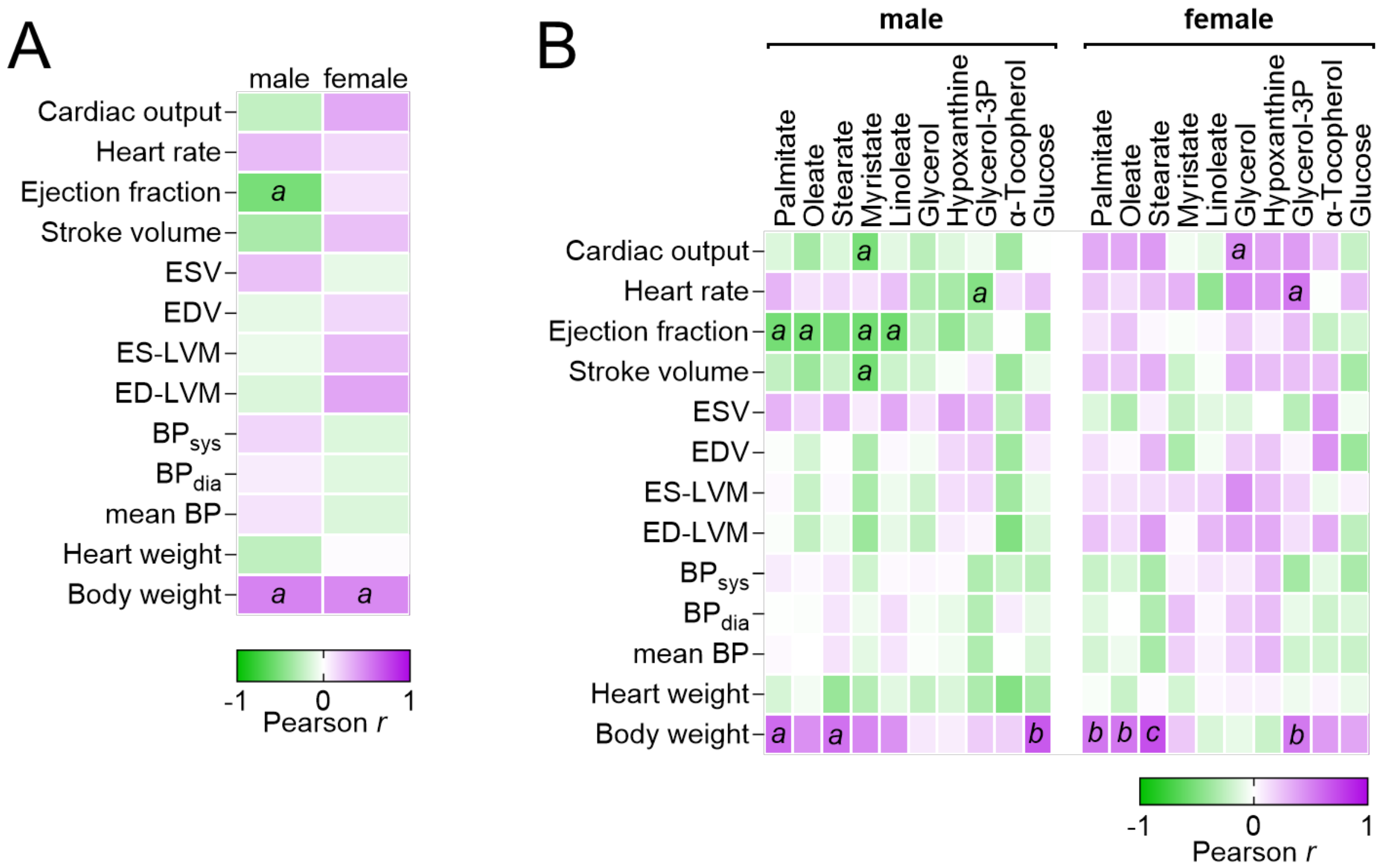

Supplement: Supplementary file 1 [file 13293_2026_1017_MOESM1_ESM.pdf]
